# UK policymaker and expert perspectives on the smoke-free generation policy: a qualitative study

**DOI:** 10.1136/bmjph-2024-001808

**Published:** 2025-02-08

**Authors:** Nathan Davies, Rachael L Murray, Tessa Langley, Joanne Morling, Manpreet Bains

**Affiliations:** 1 Nottingham Centre for Public Health and Epidemiology, University of Nottingham, Nottingham, UK; 2 SPECTRUM Consortium, Edinburgh, UK; 3 NIHR Nottingham Biomedical Research Centre, Nottingham, UK

**Keywords:** Public Health, legislation and jurisprudence, Qualitative Research, Primary Prevention

## Abstract

**Introduction:**

The UK smoke-free generation (SFG) proposal seeks to ban the sale of tobacco products to those born in or after 2009. There is substantial evidence for the benefits of raising the age of sale of tobacco but, despite several governments proposing SFG, the policy has faced significant challenge and has not been implemented at nation-state level. This study explores the context in which UK may be the first country to introduce SFG, identifies potential barriers and facilitators to SFG implementation and outlines possible approaches to SFG policy design.

**Methods:**

We conducted 19 qualitative semistructured interviews with policymakers and health leaders in England, Scotland and Wales, including politicians, public health experts, academics, trading standards experts (responsible in UK for enforcing age restrictions on products), clinicians and civil society (charity sector) representatives. Data were analysed through Kingdon’s three policy streams (problem, policy and political) and organised using the framework approach.

**Results:**

Participants conceptualised SFG as both addressing youth tobacco initiation and shifting societal norms. They agreed that all tobacco products should be included but had differing views on including e-cigarettes. Opinions on enforcement varied. Some believed minimal enforcement would suffice due to anticipated compliance, while others stressed the need for strong enforcement. All agreed enforcement should target retailers, not individuals. Politically, participants noted the rapid shift from advocates supporting Tobacco 21 to embracing SFG after government endorsement. Cohesive public health advocacy, maintaining cross-party support and public opinion and developing broader tobacco control policies were considerations for successful implementation.

**Conclusions:**

Widespread support for SFG across expert, political and public opinion provides a strong foundation for its passing into law. UK public health actors swiftly took advantage of the opening of a tobacco control policy window. Those implementing SFG must carefully consider product coverage and its approach to enforcement.

**WHAT IS ALREADY KNOWN ON THIS TOPIC**

- In New Zealand, the smoke-free generation (SFG) policy has been the focus of modelling studies, study of public support and perceptions and research into tobacco industry response. However, there is little evidence on why a SFG policy came to be announced in UK and how it should be tailored to the UK context.

**WHAT THIS STUDY ADDS**

- UK appeared to be a relatively unlikely place to lead the first country-level SFG policy, but a policy window unexpectedly opened in the lead-up to a general election. General expert consensus, strong cross-party political support, public support, low rates of youth smoking and learning lessons from SFG trailblazers are factors that could create conditions for politically viable SFG policies.

**HOW THIS STUDY MIGHT AFFECT RESEARCH, PRACTICE OR POLICY**

- Consistent and well-funded enforcement efforts will likely enhance SFG effectiveness. Policy experts should balance advocating for robust enforcement without portraying implementation as overly complex, advocate for avoiding penalising individuals for purchasing tobacco and consider staggered SFG coverage of e-cigarettes.

## Introduction

 Implementing population-level public health policy is complex^1^ and often politically controversial.^2^ New legislation and restrictions on profitable products that are likely to reduce death and disease are consistently challenged and undermined by powerful transnational actors who prioritise commercial interests over population health.^3^

The smoke-free generation (SFG) policy, also known as a tobacco-free generation policy, is a case in point. SFG consists of a ban on the sale of tobacco products and/or electronic cigarettes (e-cigarettes) to anyone born after a specific year, effectively raising the age of sale of tobacco by 1 year every year.^4 5^ The chief rationale of the SFG policy is to prevent initiation in children by removing smoking as a state-sanctioned rite of passage into adulthood.^5^ This in turn can start a ‘norm cascade’, whereby smoking becomes rarer and rarer among young adults, which in turn changes social norms and reduces the peer influence to start smoking.^4^ Its proponents also suggest it could have a ‘demonstration effect’, in which SFG sends a strong signal to adults that society is heading towards smoke-free status, potentially increasing quit rates.^5^

Empirically, there is evidence that raising the age of sale from 16 to 18^6^ and from 18 to 21^7^ reduces cigarette use in target populations. Modelling studies for SFG implementation in Singapore,^8 9^ New Zealand Aotearoa,^10 11^ the Solomon Islands^12^ and the UK^13^ project that SFG will make substantial contributions to achieving the tobacco endgame, where tobacco harm in society approaches zero.

Several jurisdictions have attempted to introduce SFG, including Malaysia,^14^ Finland,^15^ Denmark^16^ and Tasmania^17^ but all have been rescinded, blocked or challenged. In February 2024, Brookline, a small town in Massachusetts, USA, successfully defended at state Supreme Court level their local bylaw introducing a nicotine-free generation (NFG) ban on tobacco sales to all those born after 2000.^18^ Other towns, including Stoneham, Wakefield, Melrose, Winchester, Malden and Reading, passed similar NFG laws soon after.^19^ Enforcement of the law in Brookline has begun, with US$300 fines issued to uncompliant retailers.^19^

Under a Labour government, New Zealand introduced The Smokefree Environments and Regulated Products (Smoked Tobacco) Amendment Act in 2022 to prohibit smoked tobacco sales to anyone born in or after 2009. The legislation also included significant requirements for denicotinisation of cigarettes and a 90% decrease in tobacco licensed retailers.^20^ Studies showed support for the policy among young people,^21^ Māori who smoke^22^ and population-wide smokers and ex-smokers.^23^ However, in 2023 the new National Party-led government announced the law would be repealed^24^ describing SFG as ‘untested’ and ‘virtue signalling’.^25^ Researchers have outlined tobacco industry approaches to disparage the bill, and highlighted possible communication between the tobacco industry and politicians.^26^

The New Zealand approach to SFG was recommended to the UK government in the independent government-commissioned Khan review in 2022.^27^ The Conservative government took up this recommendation and announced an SFG policy^28^ to be introduced to Parliament through the Tobacco and Vapes Bill in March 2024.^29^ It passed through the early stages of the legislative process with cross-party support, but it failed to become a law before the dissolution of Parliament for a general election campaign in May 2024.^30^ Labour party, who formed the new UK government following the July 2024 election, have committed to introducing SFG.^31^ There is widespread public support for SFG^32^ and its aims resonate with young people.^33^

However, experiences from New Zealand and other nations suggest that any legislation could yet face major opposition before implementation. Furthermore, if the SFG policy is introduced, because of its novelty, there will be no direct empirical evidence from other nations to inform its design or enforcement. This qualitative study, comprised of interviews with tobacco control decision-makers and experts, seeks to address three key questions on the UK’s potential implementation of SFG:

1. What are the contextual factors that may contribute to UK becoming the first nation to implement a SFG policy?
2. What are the barriers and facilitators for SFG being carried through to enactment?
3. If SFG is enacted, how could its effectiveness be maximised and its negative consequences minimised?

## Methods

### Design and epistemology

The research took a constructionist approach,^34^ meaning that participant contributions are not just taken at face value but ascribed meaning and interpreted through the prism of Kingdon’s multiple policy streams framework,^35^ a well-established approach to analysing policymaking through the broad areas of problem, policy and politics. Bracketing—a process of recognising, writing down and setting aside pre-existing researcher views on a topic and interviewees^36^—was used to minimise researcher influence across the study process to ensure that data collection, interpretation and reporting is free from bias. The Consolidated criteria for Reporting Qualitative research framework was used to strengthen the trustworthiness of findings.^37^

### Sampling and recruitment

Policymakers and experts in England, Wales and Scotland, along with international academics with the power to inform, influence or make decisions over age-of-sale policy were identified through discussions with UK advocates, governmental leaders, and academic leaders in tobacco control.

We approached 35 individuals for interviews via email, with 16 individuals not responding, turning down interviews based on lack of time, or suggesting other participants who they felt were better placed to comment. Our final sample comprised 19 politicians, public health leaders, tobacco control researchers, trading standards experts (responsible in UK for enforcing age restrictions on products), clinicians, international experts, civil society leaders (charity sector leaders) and one retailer membership organisation that counted tobacco companies among its membership. Participants were approached based on experience, expertise and diversity of role and location and provided with a study information sheet.

### Data collection

An interview topic guide was developed based on age-of-sale literature, the UK government’s SFG policy paper,^28^ the Tobacco 21 laws introduced in the USA,^7^ and New Zealand’s approach to SFG^20^ (online supplemental file 1).

Semistructured interviews were conducted by trained qualitative researcher, ND, and recorded over Microsoft Teams between November 2023 and March 2024, lasting between 22 min and 74 min with a median duration of 40 min. Initial transcription was made through the University of Nottingham’s automated transcription service and corrections were made by ND. Recruitment was stopped when saturation of responses was judged to have been met and interviews did not lead to new codes being generated.^38^

### Analysis

An inductive open coding process was initially used by ND to describe transcripts line by line to develop initial codes with use of NVivo software.^39^ The framework method was then used to deductively chart and interpret codes through the multiple policy streams framework.^35^ Related codes were collapsed together and renamed to form subthemes. Several transcripts were double-coded by MB and findings discussed iteratively throughout the analytical process to provide triangulation and support credibility.^40^ The overarching framework and additional quotes are provided in online supplemental file 2.

### Patient and public involvement

Three groups of young members of the public aged 12–21 (n=18) provided advice on the study, advising on research questions and dissemination plans.

## Results

Nineteen individuals were interviewed (table 1). Most participants had between 10 and 29 years of tobacco control experience and there was an even gender balance. England, Scotland and Wales were represented, as well as those working on a UK or international footprint.

**Table 1:** Participant characteristics

Characteristics	N
Gender	
Female	11
Males	8
Tobacco control experience (years)	
0–9	4
10–19	7
20–29	5
30+	3
Geography covered	
England	7
Wales	2
Scotland	3
UK	5
International	2
Professional group	
Academic	2
Clinician	1
National politician	3
Third sector	4
Trading standards	3
Public health specialist	5
Retail	1

Data were themed by Kingdon’s three policy streams (problem, policy and political)^35^ and each of the three streams is subdivided into subthemes (table 2).

**Table 2:** Themes and subthemes from policymakers and expert interviews

Theme	Subtheme
Problem stream	Reducing smoking in the entire population
	Problem products
Policy stream	Approaching enforcement
	Financing enforcement
	Illicit tobacco
	No silver bullet
Political stream	Entering the policy window
	Enacting SFG

SFG, smoke-free generation.

### Problem stream

This section analyses how participants conceptualised the ‘problem’ to which SFG may be part of the solution.

#### Reducing smoking in the entire population

The risk of young people continuing to be harmed by tobacco addiction and tobacco-related diseases was a clear problem statement outlined by most participants. Participants powerfully and clearly linked this problem to the SFG policy; one interviewee said, ‘*This is to stop the next generation of children from starting up a deadly habit*’ (Civil society professional, England).

Some also conceptualised SFG as tackling a significantly broader problem; societal portrayal of tobacco as a normal consumer product for adults in UK. To these participants, SFG could support the radical reconfiguration of tobacco as a product that is never acceptable to be sold for human consumption at any age, and thus shift smoking norms across all age groups. This grouping conceptualised SFG as a policy able to send a far stronger denormalisation message than Tobacco 21; one participant described Tobacco 21 as ‘*implying that you are accepting that people should be allowed to kind of smoke and that it’s a choice*’ (Public health specialist, Scotland).

#### Problem products

Defining which tobacco and nicotine-based products are priority public health issues is the subject of some debate. However, there was consensus across nearly all participants that all tobacco products produced net harms and thus should be covered by SFG; for example, one participant noted ‘*Pan and bidi and cigarillos and things may not be adequately captured, and I think we need to make sure that we are as comprehensive as possible with whatever the legislation ends up being*’ (Civil society leader, Scotland). Participants recognised the tobacco industry may seek exemption for heat-not-burn products and other products.

There was significant divergence on the problem posed by youth e-cigarette use. In general, Scottish policymakers and experts were more likely to view e-cigarettes as an overall threat to health, not a boon, and so supported its inclusion in SFG. One international expert also held this view. English and Welsh participants, however, broadly favoured tackling youth e-cigarette use separately. Views varied, with one participant seeing e-cigarette policy as a potential distraction, a debate that could ‘*scare the horses*’ and result in ‘*losing that big prize*’ of SFG (Public health specialist, England). Others advocated for e-cigarettes to be included in SFG once youth tobacco rates are close to zero. Many raised potential harms of current smokers equivalising harm from e-cigarettes and tobacco if SFG was introduced simultaneously for both products.

### Policy stream

This section analyses participant views on the form SFG should take and how it should be implemented.

#### Approaching enforcement

There was a plurality of views on the difficulty and necessity of close enforcement of the new legislation. Some public health professionals, civil society leaders and trading standards members suggested enforcement would only need to be minimal, citing low youth tobacco use rates and success in large-scale adherence to smoke-free places legislation despite limited enforcement; as one participant said, ‘*I suspect it will be a bit like smoking in public places that, almost before it’s put into effect, people will accept it*’ (Public health specialist, England).

Others suggested a strong, early enforcement effort would be required to ensure the law was seen as meaningful. The retail association participant supported strong focus on proxy sales; trading standards participants, however, described the very high degree of difficulty of prosecuting proxy sales.

Several participants expressed the necessity of clear, regular communication to small retailers before implementation, due to possible difficulties reading complex written English. One participant perceived there to have been a paucity of communication during the last age-of-sale rise from 16 to 18 that required remedying in SFG. Others proposed that SFG would be simpler to implement than Tobacco 21, noting a single sign with ‘Tobacco can only be sold to those born before 2009’ can be left up indefinitely, and no jump is required in age limit.

There was near-total consensus among participants that individual members of the public should not be penalised for seeking to purchase tobacco, and the retailer alone should face sanction, based on the significant damage criminalising children could cause. This was put strongly; for example, ‘*I wouldn't want to criminalise kids. I think that would be a terrible thing to do*’ (Public health specialist, England).

#### Financing enforcement

Participants cautiously welcomed funding promises for enforcement teams from the UK government. However, several trading standards and public health experts placed a strong emphasis on disbursement mechanisms, warning that trading standards funds earmarked for tobacco enforcement were often diverted to shore up underfunded local authority budgets. Some participants displayed a degree of fatalism on how this could be tackled in practice, describing trading standards as a ‘*tiny pimple on the surface of local authority*’ (Trading Standards professional, England).

#### Illicit tobacco

The interaction between SFG and illicit tobacco (IT) was perceived in several different ways. A grouping of public health specialists, civil society leaders and academics highlighted that raising the spectre of IT was a well-worn tobacco industry tactic to challenge regulation, and stated the greatest influence over IT was enforcement strategy and funding, not regulation. One participant said, ‘*If the enforcement strategy at the border internationally and at retail level continues to be effective and strong, there’s no reason to think that (SFG) would increase the sale of illicit tobacco*’ (Civil society leader, England).

Some trading standards officers and politicians predicted there would be a degree of additional illicit activity caused by SFG. However, when probed, none thought this would substantially impact their assessment of the value of SFG in reducing smoking rates.

### No silver bullet

Although many participants described SFG as a holistic policy that addressed both youth smoking and societal norms around tobacco, few considered it to be sufficient alone. Many pointed towards the long time horizon for SFG impact to signify a requirement for further tobacco control policies. UK-based participants suggested additional policies such as a levy on the tobacco industry and increased focus on smoking cessation, building on political momentum generated through SFG. International experts noted New Zealand’s plan relied on licensing and denicotinisation for early projected falls in prevalence, absent from UK policy.

UK-based participants were generally supportive of introducing licensing, including those who would enforce it, who had ‘*come round to the idea…it could make a massive difference*’ (Trading Standards professional, Scotland). There appeared to be an underlying assumption from some participants that UK could not move to a New Zealand-style licence reduction ‘in one step’ without first establishing a licensing programme.

One politician opposed a licensing system on the basis it would penalise rural areas, and a retail representative queried whether any licensing would be cost-effective, citing problems with alcohol licenses.

### Political factors

This section examines the political landscape and factors influencing the emergence and advancement of SFG.

#### Entering the policy window

SFG appeared to have been viewed as originally unachievable by many UK tobacco control experts before the UK government announced their commitment to the policy. New Zealand’s SFG policy was previously seen as ‘New Zealand exceptionalism’, with parallels drawn with the uniquely robust New Zealand COVID-19 response. Many public health specialists and civil society leaders had originally supported the more ‘realistic’ Smokefree Action Coalition goal of Tobacco 21. However, following the UK SFG announcement, participants described being quickly convinced by SFG, particularly its message that tobacco is unacceptably dangerous and should not be tolerated as a consumer product. ‘*I was certainly in support of the increase to 21 but on reflection I think…this is really crucial, probably more for the symbolic act on tobacco and acting on tobacco than anything else*’ (Public health specialist, England).

Other participants, often with less experience of tobacco control, had not formed a view on Tobacco 21, but supported the SFG approach to protecting young people from addiction and harm and reducing inequalities. One participant, from a trade association body, maintained their organisation was neutral on effectiveness of public health policy and merely commented on impact on business.

Participants suggested several reasons behind the timing of the UK government announcement of SFG. This included the significant groundwork laid by the Smokefree Action Coalition, a group of charities, medical organisations and public health bodies of Tobacco 21. This group policy published several evidence-based briefing documents on Tobacco 21, supported an all-party parliamentary group to develop recommendations and commissioned public polling, which showed support for an increased age for sale of tobacco across all demographics. Although Tobacco 21 is a distinct policy to SFG, interviewees indicated that work on this helped normalise the idea that political conditions were right for introducing new age-of-sale legislation. As one participant said, ‘*We just do have this really, really strong cross-party consensus and that goes back to the last two decades of campaigning*’ (Civil society leader, England).

Further contributors included the SFG policy trailblazed by New Zealand, the subsequent recommendation of introducing SFG in the government-commissioned independent report by Javed Khan, and the role of England’s Chief Medical Officer in pressing for age-of-sale laws when the Conservative government was looking for big policy ideas. The simplicity of the policy appealed to political interviewees.

Separately, the rise in UK youth vaping was viewed as a necessary component to the introduction of SFG by several participants. Public concern about vaping had put nicotine-based products high on the Prime Minister’s immediate agenda, which opened the door to conversations about tobacco and SFG. The role of vapes in securing cross-party support was articulated by a participant, who, referring to SFG’s inclusion in the wider Tobacco and Vapes Bill, said, ‘*What would help it go through, interestingly, is tying it to the vapes for me. It makes me more likely to back it, because on its own, would I necessarily want to pursue a policy of banning people from buying cigarettes and they're born in 2009 or later? Potentially not*’ (Conservative politician, UK).

#### Enacting SFG

There was a divergence of views on how likely SFG was to pass into law and be implemented. Some public health specialists referenced the perceived role of the tobacco industry and associates in influencing the rollback of New Zealand’s SFG legislation. Some predicted UK political support would be fairly fragile; a minority were downbeat about the strength of public health voices in the media, such as the participant who cautioned, ‘*we're quite good at talking to each other, and we probably ought to be talking more broadly to the public*’ (Public health specialist, England).

Others, including public health specialists and civil society leaders, argued there was strong cross-party support, a strong health advocacy voice and a public largely supportive of towards SFG, and this meant chances of success were high. A participant reported, ‘*We … have so much evidence - around 70% of the public support the age of sale legislation and only 14% oppose it. But I don't think you see that coming through in the media because it’s a positive story and that doesn't tend to take the headlines*’ (Civil society leader, UK).

All political participants suggested tobacco was not an issue of great importance to most members of the public, although this did not appear to be perceived as a huge barrier to the legislation passing. Participants noted that the media environment on tobacco control measures had significantly changed, from strong opposition in the past to a small minority of public adversaries. Most participants agreed a strong, loud, cohesive public health voice on SFG alongside continued demonstration of public support would strengthen the likelihood of legislation being enacted.

## Discussion

Our findings show a rapid policy expert shift from supporting Tobacco 21 to endorsing SFG, and mobilisation of public health advocacy, cross-party political support and public opinion that was built on strong tobacco control advocacy foundations. Key design considerations include comprehensive product coverage and the role of SFG as a catalyst for broader tobacco control measures, although views on including e-cigarettes were mixed. Proposed enforcement strategies varied, with suggestions ranging from minimal intervention to significant early efforts.

Cross-party political support and public opinion emerged as significant factors behind the UK SFG announcement, highlighting the necessity of policy stream alignment. There was widespread emphasis of the central role of the Smokefree Action Coalition in building long-term cross-party political support over decades. Interviewees expressed extreme wariness of tobacco industry interference, concerns heightened by the experience of New Zealand’s SFG policy. In New Zealand, tobacco industry interference may have influenced the policy’s repeal through provoking fears of increased crime and loss of tax revenue, among other arguments.^41^ There is emerging evidence of tobacco industry lobbying against SFG in UK.^42^ As interviewees noted, briefing politicians and officials in non-health related departments, such as business and economy, on the Article 5.3 of WHO FCTC agreement on avoiding engagement with the tobacco industry^43^ may protect against industry interference.

Interviewees reported that broad consensus among political parties for tobacco control, built over decades, may now serve as protection for policies like SFG. The example of Brookline, USA, is instructive, with significant legal battles were fought and won against SFG opponents.^19^ This approach enables legal challenges to be contained to that local law and resolved incrementally, enabling public health specialists to build momentum that can expand regionally.^44^ A localised strategy is be less applicable to highly centralised states like UK^45^ which must pursue national-level policies directly; however, the strong focus on legal preparedness in Brookline highlights the benefits of meticulous preparation for legal challenges and careful drafting of laws.

The relative simplicity of the policy was welcomed by political interviewees, but these were among the most hesitant to consider licensing or other policies. The repealed three-strand approach in New Zealand included SFG, a 90% reduction in licensed retailers and denicotinisation of cigarettes, all of which were modelled to contribute to significant falls in smoking prevalence.^11^ As some participants remarked, political attention on tobacco may be a precious commodity in endgame scenarios where smoking prevalence is low. This suggests that in settings where smoking prevalence is low and tobacco control efforts are nearing the endgame stage, the opening of a policy window may offer a singular opportunity to introduce multiple measures, such as licensing and outlet reduction. Licensing may be particularly advantageous in enforcing SFG.^46^

Our study also highlights numerous considerations surrounding product coverage of SFG. The debate among participants about including e-cigarettes in the SFG policy reflects broader tensions in UK. In the USA—where vaping is broadly viewed as a greater public health risk^47^—nicotine-free generation policies that encompass e-cigarettes have been passed.^19 44^ For jurisdictions contemplating SFG, advocates and policymakers must carefully assess the local context of tobacco and e-cigarette use and regulatory environments to determine approach to product coverage.^46^ Staggered approaches that first cover tobacco and later cover e-cigarettes when smoking rates reach a prespecified low point may offer a way forward in some jurisdictions.

Enforcement was another polarising topic, with participants expressing divergent views on the necessity and intensity of enforcement efforts. Some advocated for minimal enforcement based on the expectation of high compliance, while others emphasised the need for robust mechanisms to ensure the policy’s effectiveness. International evidence shows that consistent enforcement is a crucial component of effective youth smoking policies^48^ and young people in UK expect and desire for SFG to be strongly enforced,^33^ suggesting low levels of enforcement may weaken policy effectiveness and acceptability in the UK context. UK expert interviewees strongly positioned themselves against penalising individual purchasers who violate SFG. This position is supported by findings from Tobacco 21 research which indicates reduced effectiveness in jurisdictions that penalised purchasers compared with those that did not.^49^

## Conclusion

The UK’s move towards implementing SFG can be attributed to alignment of public health advocacy, longstanding political consensus and public support. Effective SFG policy design will encompass comprehensive tobacco product coverage and carefully consider e-cigarette regulation dependent on local context. Well-planned enforcement strategies are crucial for policy success. We find that the relative simplicity of the policy is attractive to policymakers. Many in UK are seeking to learn from the experiences of New Zealand by maintaining sustained advocacy and protecting against industry interference. As other jurisdictions consider similar initiatives, the UK’s approach offers valuable insights into advancing and securing novel public health policies through strong political and public backing.

## Supplementary material

10.1136/bmjph-2024-001808 online supplemental file 1

10.1136/bmjph-2024-001808 online supplemental file 2

## Data Availability

Data may be obtained from a third party and are not publicly available.
