# Supplementary material for: UK policymaker and expert perspectives on the smoke-free generation policy: a qualitative study
Source: BMJ Public Health. 2025 Feb 8;3(1):e001808. doi: 10.1136/bmjph-2024-001808 (PMC11816585; doi:10.1136/bmjph-2024-001808)
Supplement: online supplemental file 1 [file bmjph-3-1-s001.pdf]

Interviews are conducted by Nathan Davies MPH, a male specialty registrar in public health and NIHR doctoral fellow studying the UK’s smokefree generation policy.

Before interview:

Explain purpose of study. Go through the participant information sheet with participant. Reiterate points about consent, confidentiality, safeguarding, recording, treatment of data and withdrawal.

Do you have any questions about any part of the study?

ROLE

- What is your current professional role or roles?
- How long have you worked on/contributed to decision-making or research on tobacco-related issues?
- What was your motivation for working on tobacco?

Age-of-sale of cigarettes and e-cigarettes

1. What is your understanding of the history of the policy and implementation of age-of-sale policy of tobacco in (England/Wales/Scotland/UK as a whole)?

Prompts: Can you describe how effective this policy has been? What are its strengths and weaknesses?

2. The UK Government, Scotland and Wales is proposing to bring forward legislation making it an offence to sell tobacco products to anyone born on or after 1 January 2009. In effect, this raises the smoking age by a year every year. What is your view on this proposal?

IF NOT COVERED: What do you think of the idea compared to:

- Raising the age of sale of tobacco to 21, as in the US or Singapore?
- Raising the age of sale of tobacco to 25, as some researchers have advocated?

3. The proposed law will raise the age of sale of most tobacco-containing products, but not vapes or e-cigarettes. What is your view on this?

4. Can you describe the international context of age-of-sale of tobacco policies? Is there any learning for the United Kingdom?

5. What sort of factors have been a facilitator or a barrier to the age of sale law being proposed?

Prompts: What about the following?

- Media environment
- Political environment
- Public perceptions
- Health organisation environment
- Advocacy environment

6. What sort of factors might be a facilitator or a barrier to the age of sale law being implemented?

Prompts: What about the following?

- Media environment
- Political environment
- Public perceptions
- Health organisation environment
- Advocacy environment

7. If it were agreed, what would need to be considered in implementing a new age-of-sale law?

Prompts: What about…

- Illicit tobacco
- Communications campaigns
- Enforcement (e.g. Trading Standards)
- Public engagement and buy-in
- Support for quitting
- Role of children and young people?

8. Are there any other policies or forms of support you think should be put in place at the same time as new laws on age of sale if they were to be implemented?

Prompts:

- Criminalising purchaser (currently in Scotland)
- Proxy purchasing
- New Zealand licensing scheme to significantly reduce the number of retail outlets that can sell tobacco
- New limits to reduce the nicotine strength of cigarettes.

9. Do you have any final points you'd like to make?

Thank you for taking part.
