# Supplementary material for: UK policymaker and expert perspectives on the smoke-free generation policy: a qualitative study
Source: BMJ Public Health. 2025 Feb 8;3(1):e001808. doi: 10.1136/bmjph-2024-001808 (PMC11816585; doi:10.1136/bmjph-2024-001808)
Supplement: online supplemental file 2 [file bmjph-3-1-s002.pdf]

Additional supporting quotes

Theme	Subtheme	Quotes
**Problem stream**	**Reducing smoking in the entire population**	"And obviously, tobacco products by any means have a significant cost to them, both economic and health wise. So I think anything to try and reduce that would be advantageous for the overall health of the population and also help to reduce health inequalities." Clinician, England
		"Which decision is less harmful, the surrender of freedoms or saying to people, pick up a fire stick full of tar and light it up and put it in your mouth. It is one of the many decisions we'll have to make." Politician, England
	**Problem products**	"I think we need to make it comprehensive. So heated tobacco products absolutely should be included. And you know, all forms of tobacco. So I think one of the issues is the minority ethnic forms of tobacco have not up till now been included in, for example, advertising bans. So pan and bidi and cigarillos and things may not be adequately captured, and I think we need to make sure that we are as comprehensive as possible with whatever the legislation ends up being." Civil society leader, Scotland
		But I think that there are steps the government can take before age of sale, right. So changing the (e-cigarette) packaging, changing the flavours, changing where they can be placed in shops before they make them inaccessible to people. Civil society leader, UK
		"I think the other thing to observe on the vaping elements of this is the difference of approach from devolved administrations, so Scotland and Wales. I think Scotland have always been more interested in going further and faster on the vaping side of things and that's going to be an interesting debate, a little bit further down the line because although this is the UK wide consultation, there's already a Scottish proposal of supposed to expand age of sale restrictions on vapes potentially in the future is as well, which is not part of the discussion in terms of the UK consultation." Civil society leader, Scotland
**Policy stream**	**Approaching enforcement**	"A lot of tobacco control policies go through with very little enforcement needed. You know, you look at smoke free itself, the original smoke free, but also display. I mean, you very rarely see tobacco and display now, do you anywhere. I mean people comply with the law and sometimes if you see a tobacco display open and you say that tobacco installation shouldn't be open, they usually close it straight away." Trading standards professional, England
		"So we're really interested in mandatory age verification. Requiring ID for all and all people purchasing tobacco which is in and of itself, would probably have an impact on people's behaviour and which would be interesting and untested, but would enable. And you know, if you think about it, you know, when everyone when the legal age is like 30, you know you need to be IDing everybody." Civil society leader, England
	**Financing enforcement**	"I think we'd like to see more enforcement. The thing we'd like to see more money for enforcement, and we feel that the enforcement side of this is actually the most important part that is sort of getting missed in this debate because we already have an age restriction on tobacco products and vaping products, but perhaps we're not seeing the level of enforcement that we would need to stop businesses." Retail representative, UK

Theme	Subtheme	Quotes
**Policy stream**	**Illicit tobacco**	“I'd just go back to the point that tobacco companies always say illicit trade will be a problem. They see that when we had large excise taxes and when we introduce plain packaging and yet over that a 10 year period of sustained policy activity, we've seen very, very stable estimates of around 5% of foreign packs. So I just don't see that it's going to be a really big problem here.” Academic, international
		“My experience of working in illicit tobacco issues in [place] when I was there is that the various partners are very happy to work together on it and that work is ongoing. It needs to be properly resourced but otherwise that's fine. We can deal with that.” Public health professional, England
		“You know, if you look at the amount of tobacco regulation we've had since just since the year 2000, it's more and more and more. We've banned vending machines, we've banned displays, we've banned standardized packaging and all this stuff, and every single time a piece of new regulation has been brought in, the tobacco industry will say it's going to lead to an increase in illicit. And it hasn't.” Trading standards professional, Scotland
	**No silver bullet**	“And so the government needs to introduce a tobacco levy. This measure was carried in the House of Lords, suddenly turned down in the Commons, nearly got through the Lords again, and it will be a way you could finance targeted advertising campaigns to reduce the problems of smoking and to support this ban and to explain to people next year, 19 year olds cannot be legally sold tobacco.” Politician, England
		“I do think that there needs to be a kind of licencing of products, tobacco as well. So there is essentially that there's more leverage in in being able to take a licence away from an individual. At the moment it's very difficult to get a prosecution”. Public health professional, England
		“We still have a quite a big illicit alcohol, non duty-paid alcohol problem and that's not because the licensing systems bad is because they don’t go out there and enforce it the same way. So I think licensing systems are in themselves and not a solution without the enforcement to back them up.” Retail representative, UK
**Political stream**	Entering the policy window	“I shouldn't underplay the impact that the that that are really well organized health lobby, health advocacy network, has had on the political process. We are able to galvanise very high levels of support across all sorts of different organisations, so you've got integrated care boards, NHS Trusts, local authorities, trading standards teams, small NGOs, big NGOs, Royal colleges, professional bodies, coming out and backing this measure, responding to the consultation and that's because we're well organised and well embedded, trusted.” Civil society leader, England
		“The thing is, it's never one thing, is it? So it's cumulation of circumstance. So you have New Zealand pursuing their policies and kind of that the international attention that that gathers obviously has obviously been very important and it's why that was a policy that was in the Khan review. If you take away New Zealand pursuing this as a policy, uh, it probably doesn't end up in the Khan review.” Civil society leader, England

Theme	Subtheme	Quotes
**Political stream**	**Entering the policy window**	“You’ve got the Chief Medical Officers backing it.” Politician, England
		“So the PM taking an interest in and probably vaping was the thing that had raised the agenda to his attention, because that's obviously been quite a high profile issue.” Civil society leader, England
	**Enacting SFG**	“So I'm gonna go all out. We all know these things are like David and Goliath, and mostly Goliath wins. Sometimes David wins, but not that often. So the odds are stacked not in our favour. We might get it and I hope we're sincerely hope we do.” Public health professional, England
		“You know, there'll be another effort from tobacco industry to try to kind of shape this. So they will look too, I think probably at age of sale rather than smoke free generation because they want to maintain some sort of industry. They might also want to try to ensure that these you know from the tobacco industry perspective, what you, the worst thing you can have is examples in places of effective bans or kind of like legislation that stops them, prevents the sale of tobacco worldwide”. Public health professional, Scotland
		“So I think getting that public support is really important because when you're trying to sway politicians, often they're driven by what's going to get them re-elected. So if you've got a policy that you know is going to be politically popular, it can be quite powerful.” Academic, international
		“It would be really good if we could just come out with a simple unified line and pat on the back government for doing this and be very supportive of it without getting distracted by the other issues that could come up.” Public health professional, England
